# Dr. Josef Arkövy

**Published:** 1892-07

**Authors:** 


### DR. JOSEF ARKÖVY.

Upon the nomination of Count Csaky, Minister of Instruction of Austria, the Emperor has appointed Dr. Josef Arkövy to be Professor of Dental Science in the University of Budapest.

This appointment is one most worthily bestowed. Dr. Arkövy has occupied a prominent position as author, teacher, and clinical instructor. His work on "Diagnosis of Dental Diseases" is one of the most thorough in dental literature, and its publication raised him at once to a first rank in his profession in Hungary and the world.

